# Intraprocedural Acute Stent Thrombosis of Unusual Etiology

**DOI:** 10.7759/cureus.91601

**Published:** 2025-09-04

**Authors:** Binayendu Prakash, Tapan Kumar, Sritam Acharya, Mandar M Shah

**Affiliations:** 1 Cardiology, Tata Main Hospital, Jamshedpur, IND

**Keywords:** acute stent thrombosis, angioplasty and stenting, coronary stents, myocardia infarction, thrombosis

## Abstract

Stent thrombosis is a rare but potentially fatal complication of percutaneous coronary intervention (PCI), typically associated with a combination of factors, such as issues with the stent itself (poor expansion, malapposition), patient-associated factors (non-compliance with antiplatelet medication, hypercoagulability), and procedural issues (vascular injury, incomplete lesion coverage).

This article presents two unique cases of acute stent thrombosis occurring within minutes of stent deployment, attributed to embolization of cotton fibers (lint) from surgical drapes and reused instruments. In both cases, thrombosuction revealed fibrous foreign material as the underlying cause. Despite the acute and severe presentation, timely intervention with repeated thrombosuction, intracoronary pharmacotherapy, and balloon dilatation successfully restored coronary flow to TIMI III flow (thrombolysis in myocardial infarction score). These cases underscore the importance of strict contamination control, the use of lint-free materials, and adherence to sterile techniques in catheterization labs. Enhanced awareness and preventive protocols can significantly reduce the incidence of such rare but serious complications.

## Introduction

Significant obstructive coronary artery diseases are mostly treated with drug-eluting coronary stents, and stent thrombosis is a major complication associated with it. Stent thrombosis is defined as a thrombotic occlusion within 5mm proximal or distal of a coronary stent leading to ischemic signs or symptoms. It leads to high rates of morbidity and mortality due to cardiac death or nonfatal myocardial infarction (MI) and often results in acute coronary syndrome (ACS)[1-3]. Risk factors include patient-related elements (e.g., diabetes mellitus, ACS, reduced ejection fraction, renal impairment), procedural aspects (e.g., stent malapposition, stent underexpansion), lack or discontinuation of dual antiplatelet therapy (DAPT), high platelet reactivity, and incomplete P2Y12 inhibition [2,3].

As of 2008, the Academic Research Consortium (ARC) guidelines classifications of stent thrombosis were based on the type of underlying stent, clinical scenario, and timing after initial stent placement [4].

Stent thrombosis is categorized by timing as: 1) acute: within 24 hours of stent placement, 2) subacute: 24 hours to 1 month, 3) early: within 1 month, 4) late: 1 to 12 months, 5) very late: after 12 months [5].

We present two rare cases of periprocedural acute stent thrombosis that occurred within minutes of stent placement, secondary to linen lint/fiber embolization, with successful management. Corrective measures to prevent recurrence are also discussed.

## Case presentation

Case 1

A 54-year-old male presented with chest discomfort persisting for two days. His medical history included type II diabetes mellitus, diagnosed five years prior. On admission, the patient was hemodynamically stable.

The electrocardiogram (ECG) demonstrated first-degree atrioventricular (AV) block. Echocardiography revealed no regional wall motion abnormalities (RWMA), and left ventricular systolic function was preserved. Laboratory investigations showed elevated troponin levels. A diagnosis of non-ST-elevation myocardial infarction (NSTEMI) was made, and he was initiated on dual antiplatelet therapy (DAPT), statins, and low molecular weight heparin (LMWH).

On coronary angiography, the right coronary artery (RCA) showed significant 90% stenosis in the mid-segment. The left anterior descending (LAD) and left circumflex (LCX) arteries showed minor lesions.

Percutaneous coronary intervention (PCI) of the RCA was planned. RCA was engaged using a JR (Judkins right) 4.0 guiding catheter. The lesion was crossed with a Whisper MS (middle support) wire. The lesion was pre-dilated with a 3.0 × 12 mm NC (non-compliant) Quantum Apex balloon at 16 atmospheres. A 3.0 × 38 mm Synergy XD (extended delivery) stent was deployed at nine atmospheres for 20 seconds. Post-dilation was performed with a 3.0 × 12 mm NC quantum apex balloon up to 18 ATM and a 3.5 × 12 mm NC quantum apex balloon. The procedure achieved a good angiographic result with TIMI III flow and no residual stenosis.

A few minutes post-procedure, the patient experienced chest discomfort. The monitor revealed atrial ectopics, and a check angiogram revealed a Grade V thrombus in the stented segment of the RCA with no distal flow.

**Figure 1:**
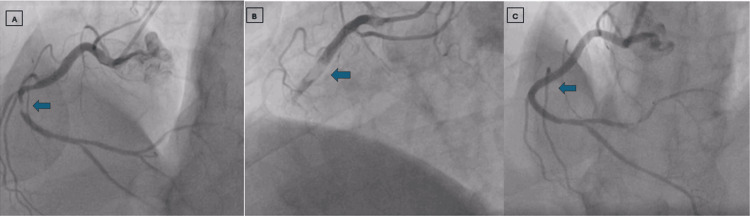
Angiographic view of RCA (right coronary artery) Figure 1A shows a significant lesion in the mid RCA. Figure 1B shows a large thrombus after stent placement. Figure 1C shows the final result after repeated thrombosuction.

RCA was rewired, and immediate thrombosuction was performed. Aspirated thrombus revealed a bit of cloth fiber. Repeated thrombosuction aspirated multiple small fragments of cloth fiber. The thrombus got dislodged and migrated to a distal vessel. A bolus of the intravenous antiplatelet agent cangrelor was administered; however, distal flow was not restored. Further thrombosuction attempts achieved TIMI I flow. Intracoronary antiplatelets, heparin, and the vasodilator agent nicorandil were administered through the export catheter. The distal flow remained TIMI I. Further, distal vessel dotter in and dilation with a 2.0 × 10 mm NC balloon was done; it improved flow to TIMI III. One branch of the posterolateral ventricular (PLV) artery showed no flow; otherwise, distal flow was satisfactory (TIMI III). Figure 1 shows the RCA lesion before and after stenting and after repeated thrombosuction.

The patient tolerated the procedure well; his vitals remained stable throughout the procedure. Post-procedure, he developed anemia, which was managed with a packed red blood transfusion. At the two-month follow-up, the patient was asymptomatic and clinically stable.

Case 2

A 74-year-old female presented with acute chest discomfort and shortness of breath for one day. ECG revealed ST depression in V1-V4 chest leads. Her troponin I value was 0.76 ng/ml. Echocardiography showed hypokinesia of the inferior-posterior wall with an LV ejection fraction of 42%.

Her angiogram showed a critical 90% stenosis of the left circumflex (LCX); other coronary arteries were normal.

The left main artery was engaged with an XB (extra backup) 3.5 guiding catheter, and the LCX to obtuse marginal branch was wired with a reused whisper coronary wire. The lesion was pre-dilated with a 3.0 x 12 mm NC balloon & a 3.0 x 15 mm OPN balloon and stented with a 3.0 x 26 mm Onyx Frontier stent. The angiographic picture after stent deployment showed a good result with distal TIMI 3 flow. Further, it was post-dilated with a 3.5 x 12 mm NC balloon. The patient suddenly developed chest discomfort with profuse sweating. The repeat angiographic picture showed a large thrombus in the left main and left circumflex, as well as in the obtuse marginal branch vessel. Immediately, six French thrombo-suction catheters were negotiated, and suctioning was done. The aspirated content showed multiple bits of greenish fiber (lint of the linen used for draping).

**Figure 2:**
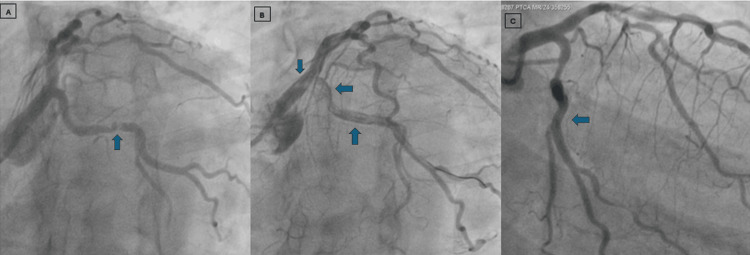
Angiographic view of LCX (left circumflex artery) Figure 2A shows a significant LCX lesion in the proximal part. Figure 2B shows a large thrombus in the left main, LCX, and OM (obtuse marginal) vessel. Figure 2C shows the final angiographic view after repeated thrombosuction.

A repeated thrombo-suction of both the circumflex and obtuse marginal branches was done. Finally, after multiple aspirations, TIMI III flow was achieved, and the patient was salvaged successfully. Figure 2 shows a significant LCX lesion before and after stent placement with a large thrombus in the left main, left circumflex, and obtuse marginal branch vessels, and after repeated thrombosuction.

**Figure 3:**
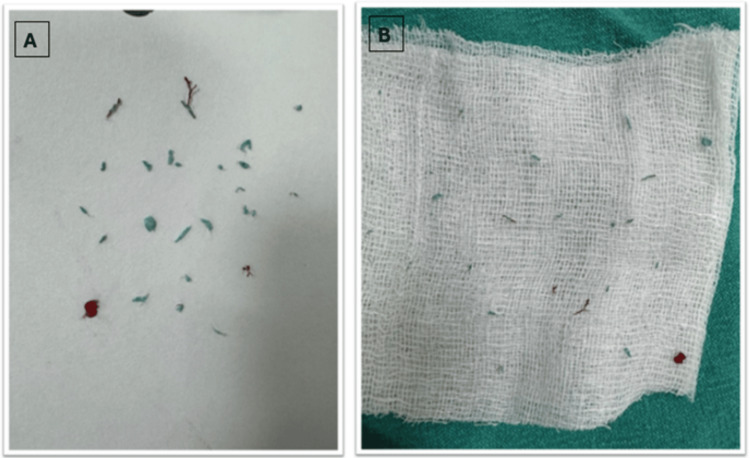
Aspirated fibers and thrombus materials Figures 3A, 3B show small pieces of aspirated fiber and thrombus materials.

Figures 3A, 3B show the aspirated fibers and thrombus material.

## Discussion

Stent thrombosis remains one of the most serious complications of coronary angioplasty, often leading to significant morbidity and mortality [6,7]. While well-known causes such as stent malapposition, patient comorbidities, and insufficient dual antiplatelet therapy (DAPT) are frequently implicated, foreign body contamination is a lesser recognized but critical contributor to this complication during interventional procedures [8,9].

Foreign body contamination involves the inadvertent introduction of particulate matter or fibers into the patient’s vasculature. Cotton fibers, a common contaminant in cardiac catheterization labs, originate from gauze, surgical drapes, and sponges frequently used during procedures [10,11]. The fibrous structure of cotton, combined with mechanical operations like wiping guidewires or catheters, increases the risk of fiber shedding. This contamination can lead to adverse events such as local thrombosis, pyrogenic reactions, granulomatous inflammation, and embolization [9,12].

In the cases presented, acute stent thrombosis occurred within minutes of stent deployment, an unusually rapid and life-threatening event. Multiple thrombosuction procedures revealed cotton fibers from the green draw sheet used during the procedure. The likely mechanism involved local hypercoagulability induced by the fibers, leading to the rapid formation of a thrombus. This highlights the importance of procedural vigilance and contamination control.

The management of acute stent thrombosis requires rapid identification and resolution. In these cases, repeated thrombosuction, low-pressure balloon dilatation, and intracoronary administration of antithrombotic agents successfully restored TIMI III flow; however, the event underscores the need for preventive strategies to mitigate foreign body contamination during interventional procedures. In our cases, the source of embolization of the lint was unclear; however, we could identify certain practices that could be potential sources for the same: 1) the balloons once used were kept covered within the sterile draw sheets; however, these sheets, often old and used multiple times in the past, had a tendency to form a significant amount of loose lint. It is possible that these lint particles inadvertently adhered to the balloon catheters. 2) The linen used to drape the patient was reused in both cases. The guidewires used were also reused. It is possible that lint from the draped linen got adhered to the guidewires that have lost their coating, making them more “sticky” [10].

Recommendations for improvement include vigilance in the sterile field, regular inspection for loose cotton fibers or particulate matter, replacement of cotton-based products, and use of synthetic or fiber-free materials for medical drapes, sponges, and wiping tools. Wet gauzes should be kept separate from saline bowls used for catheter flushing.

Staff training should be done to emphasize the risks associated with foreign body contamination, and staff should be trained on preventive measures [9].

Such measures can significantly reduce the incidence of contamination-related complications, ensuring safer outcomes for patients undergoing interventional procedures.

## Conclusions

Acute stent thrombosis is a rare but serious complication that demands prompt recognition and management. These cases highlight the often-overlooked risk of cotton fiber contamination during cardiac catheterization procedures. Cotton fibers, when introduced into the vasculature, can induce thrombus formation and subsequent thromboembolism, resulting in life-threatening complications.

Adopting fiber-free materials and strict contamination control measures are crucial steps to mitigate these risks. The use of sterile, biocompatible foam-based wipers and enhanced procedural protocols can minimize the introduction of particulate matter during interventions. By addressing these risks proactively, healthcare teams can improve patient safety and procedural outcomes in interventional cardiology.
